# Platelet-Derived Growth Factor Receptor Alpha in Male Reproductive Health and Oxidative Stress: Stromal Homeostasis, Injury, and Research Perspectives

**DOI:** 10.3390/antiox15091208

**Published:** 2026-09-20

**Authors:** Eun-A Ko, Jeong Seok Hwa, Dawon Kang

**Affiliations:** 1 Department of Physiology, College of Medicine, Jeju National University, Jeju 63243, Republic of Korea; koeuna@jejunu.ac.kr; 2 Department of Urology, Gyeongsang National University Hospital, Jinju 52727, Republic of Korea; 3 Department of Urology, College of Medicine, Gyeongsang National University, Jinju 52727, Republic of Korea; 4 Institute of Medical Sciences, Gyeongsang National University, Jinju 52727, Republic of Korea; 5 Department of Physiology, College of Medicine, Gyeongsang National University, Jinju 52727, Republic of Korea; 6 Department of Convergence Medical Science, Gyeongsang National University, Jinju 52727, Republic of Korea

**Keywords:** oxidative stress, PDGFRα, stromal cell, Leydig cell, spermatogenesis, male infertility

## Abstract

Oxidative stress is a well-recognized contributor to male reproductive dysfunction, yet discussion of this mechanism has focused almost exclusively on sperm damage. This focus overlooks the somatic and stromal networks that support testicular development, steroidogenesis, tissue architecture, and erectile function. Platelet-derived growth factor receptor alpha (PDGFRα) is expressed in heterogeneous interstitial and progenitor-enriched populations and has established roles in testicular development. This narrative review examines the evidence on PDGFRα in male reproductive tissues and considers the potential relevance of redox mechanisms identified predominantly in other organ systems. Genetic studies show that *Pdgfa* deficiency impairs the postnatal establishment of the adult Leydig-cell population, whereas *Pdgfra* deficiency disrupts fetal testis cord organization and Leydig-cell differentiation. Studies of the adult testis document receptor localization and, in cell-based models, downstream signaling capacity. Preclinical work has further identified PDGFRα-positive cavernosal fibroblasts associated with vascular remodeling in erectile dysfunction, although a receptor-specific function for these cells remains unproven. We propose that PDGFR-associated redox signaling may shape stromal responses to injury in the testis and penis; however, direct evidence for this mechanism in male reproductive tissue is currently lacking. Testing this hypothesis will require cell-resolved measurements of receptor phosphorylation and localized reactive oxygen species, together with lineage-restricted, receptor-specific perturbation. Establishing whether such a pathway exists could open a mechanistic link between oxidative stress and stromal, rather than purely germ-cell, contributions to male reproductive dysfunction—with implications for both testicular endocrine failure and vasculogenic erectile dysfunction.

## 1. Introduction

Reactive oxygen species (ROS) have a dual role in male reproduction. Controlled ROS production contributes to sperm capacitation, hyperactivation, acrosomal exocytosis, and sperm–oocyte interaction, whereas ROS in excess of antioxidant capacity induces lipid peroxidation, protein oxidation, mitochondrial dysfunction, and DNA damage [1,2,3,4]. This biological duality helps explain why generalized antioxidant treatment has produced inconsistent clinical outcomes: the relevant question is not simply whether ROS should be eliminated, but where, when, and to what extent redox signaling should be modified.

Most discussions of male reproductive oxidative stress focus on mature spermatozoa. This emphasis is understandable because sperm membranes are enriched in polyunsaturated fatty acids, spermatozoa possess limited cytoplasmic antioxidant reserves, and oxidative injury can be quantified through motility, lipid peroxidation, and DNA fragmentation assays [1,2,3]. Nevertheless, sperm quality is the endpoint of a much broader physiological system. Germ-cell development depends on the integrity of the seminiferous epithelium, Sertoli-cell metabolic and structural support, Leydig-cell steroidogenesis, peritubular contractility, microvascular function, and controlled remodeling of the interstitial extracellular matrix. Oxidative disruption of any of these compartments can impair fertility even when direct sperm oxidation is not the initiating event.

This distinction matters for therapeutic strategy. Clinical evidence regarding antioxidant supplementation for male infertility remains inconclusive. The updated Cochrane review identified possible improvements in live birth and clinical pregnancy. Still, the certainty of the evidence was very low, and the apparent benefit was not robust to sensitivity analyses [5]. Consistent with this uncertainty, the AUA/ASRM guideline considers the clinical utility of antioxidant supplements questionable, whereas the EAU guideline advises against their routine use in men with idiopathic infertility [6,7]. This clinical uncertainty challenges a purely damage-centered assumption that greater antioxidant exposure should uniformly improve reproductive outcomes. A somatic-niche framework offers a mechanistic hypothesis: if physiologically regulated receptor-associated redox signaling contributes to cell survival, differentiation, or repair in Leydig-lineage, peritubular, or vascular compartments, then indiscriminate suppression of ROS could attenuate physiological signaling in these populations even while limiting oxidative damage to mature spermatozoa. More broadly, ROS exerts both physiological and pathological effects in male reproduction, and maintenance of redox equilibrium is required for normal sperm function [8]. Excessive or poorly targeted antioxidant intervention may also induce reductive stress and impair redox-dependent physiological processes [9,10,11,12]. Framing oxidative stress as a somatic-niche disorder, therefore, does not simply add another cell type to consider; it reframes the therapeutic question from how much ROS should be removed to which ROS, in which cell, at which time, should be modulated.

A testicular-niche perspective is particularly relevant to aging, obesity, diabetes, heat exposure, varicocele, infection, environmental toxicants, chemotherapy, and radiation. These stressors generate overlapping combinations of mitochondrial dysfunction, inflammatory signaling, altered endocrine function, and tissue remodeling [3,4,13]. A molecular system capable of linking redox status to somatic-cell survival, differentiation, and matrix responses could therefore influence whether an oxidative insult is resolved or progresses to reproductive dysfunction.

**Figure 1:**
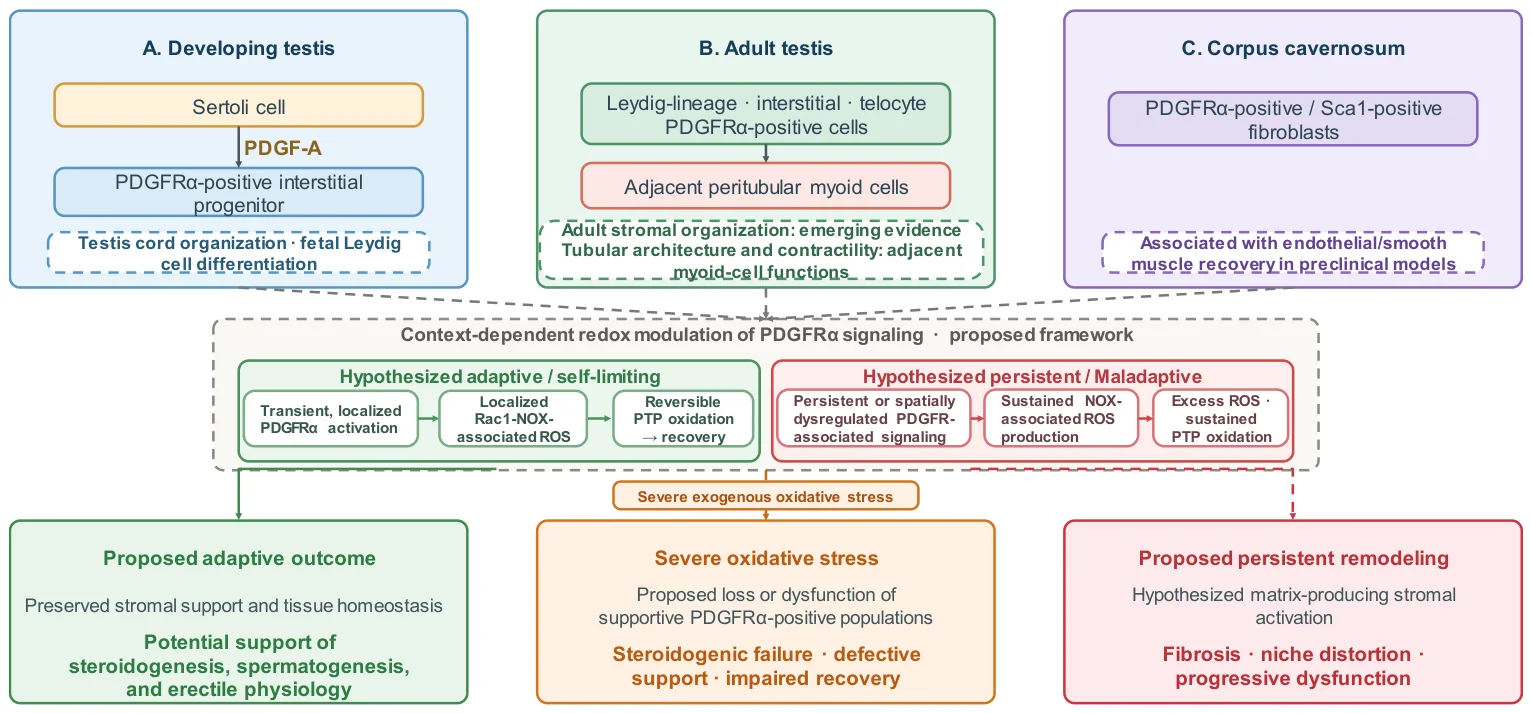
Established reproductive-tissue findings and proposed context-dependent redox roles of PDGFRα-positive stromal populations. Established or emerging reproductive-tissue findings are summarized for the developing testis, adult testis, and corpus cavernosum. The proposed framework distinguishes potentially adaptive, severe-injury, and persistent-remodeling outcomes of PDGFR-associated redox signaling. The redox–PTP–NOX relationships are extrapolated primarily from non-reproductive systems; the relevant NOX isoform, Rac1 dependence, and PDGFRα specificity remain unestablished in male reproductive tissues. Connectors indicate directional relationships, whereas evidence status is specified within each module. PDGF-A, platelet-derived growth factor A; PDGFRα, platelet-derived growth factor receptor α; NOX, NADPH oxidase; PTP, protein tyrosine phosphatase; ROS, reactive oxygen species.

The platelet-derived growth factor (PDGF)–PDGF receptor (PDGFR) system is a plausible but underexplored candidate for this role. PDGFRα signaling has established functions in fetal testis development and Leydig-lineage specification, whereas its physiological functions in the adult testis remain incompletely defined [14,15,16,17]. Separately, studies conducted predominantly in non-reproductive cells and tissues have demonstrated reciprocal interactions between PDGFR activation and ROS generation [18,19,20]. Here, we integrate these distinct bodies of evidence and propose that the PDGF–PDGFR axis may act as a redox-sensitive regulator of the testicular somatic-cell niche (Figure 1). This proposed testicular PDGFR–ROS axis is a working hypothesis and should not be interpreted as an established mechanism. Rather, we identify evidence supporting its biological plausibility and define the experiments needed to determine whether PDGFR-associated signaling is adaptive or maladaptive in specific male reproductive contexts. Accordingly, this article is presented as a narrative review: it synthesizes evidence from heterogeneous experimental systems, including human and rodent reproductive tissues and non-reproductive models used explicitly for mechanistic extrapolation, rather than providing a preregistered systematic evaluation of a homogeneous evidence base. Throughout the review, we distinguish direct reproductive-tissue evidence from reproductive cell-based observations, nonspecific pharmacological findings, mechanisms established in non-reproductive models, and hypotheses requiring direct validation.

## 2. Methods

### Search Strategy and Study Selection

This review was conducted as a narrative synthesis of experimental evidence on PDGFRα expression, regulation, and function in the male reproductive system and on its proposed relationship to the cellular redox state. Database searches were finalized on 30 June 2026. Electronic searches combined the terms “PDGFRA”, “PDGFR-alpha”, “PDGFR alpha”, “platelet-derived growth factor receptor alpha”, and “CD140a” with “testis”, “testicular”, “Leydig cell”, “Sertoli cell”, “peritubular myoid cell”, “seminiferous tubule”, “epididymis”, “corpus cavernosum”, “male infertility”, “male fertility”, “male reproductive”, “spermatogenesis”, “oxidative stress”, “reactive oxygen species”, and “fibrosis”.

The database-specific searches yielded 154 records from a title- and abstract-restricted Lens.org search, 332 records from a title- and abstract-based PubMed search, and 514 records from a Topic search of the Web of Science Core Collection—the Web of Science search covered records indexed from 1 January 1990 to 30 June 2026. The PubMed search was restricted to publications dated through 30 June 2026; records electronically published by that date were retained even if they were subsequently assigned to a later print issue. These numbers represent the raw database-specific yields, and overlap among databases was expected. Google Scholar was used for supplementary searching and citation tracking, but was excluded from the formal record count because its estimated result totals are not stable or fully reproducible. Reference lists of the core literature were examined to identify additional primary studies not captured by the structured searches and to retrieve relevant historical and general mechanistic background sources. Because retrieval routes were not recorded prospectively at the level of individual references, and many articles were identified through more than one source, references could not be reliably assigned to a single discovery route.

Studies were included if they reported expression, regulation, or functional data on PDGF/PDGFR signaling in mammalian male reproductive tissues at any developmental stage, or if they reported redox–PDGFR interactions in non-reproductive tissues that were used explicitly as extrapolated mechanistic evidence and identified as such in the text. No study was excluded solely based on publication year. Studies published before the documented Web of Science interval remained eligible and could be retrieved through PubMed, Lens.org, or backward citation searching. Studies focused exclusively on PDGFRA as an oncological biomarker, including gastrointestinal stromal tumors, prostate cancer stroma, and testicular germcell tumor genomics, were excluded unless they also provided male reproductive tissue data relevant to the present synthesis.

As a narrative review, this synthesis did not follow a PRISMA-based systematic review workflow, and no formal risk-of-bias assessment was performed. The possibility of selection bias is therefore acknowledged as a limitation of the narrative approach. The literature identified through this approach reveals a progression from descriptive mapping of PDGF ligands and receptors to causal studies of testicular development and, more recently, to single-cell and functional analyses of stromal-cell-mediated tissue repair.

## 3. Physiological Roles of PDGF-PDGFR Signaling in Male Reproductive Tissues

### 3.1. Testicular Development and the Leydig-Cell Lineage

The PDGF family comprises five dimeric ligands—PDGF-AA-, PDGF-AB, PDGF-BB, PDGF-CC, and PDGF-DD—formed from four polypeptide chains encoded by PDGFA, PDGFB, PDGFC, and PDGFD. Northern blot analysis detected *Pdgfc* transcripts in the adult mouse testis, including an additional 1.8-kb transcript not reported in the other tissues examined [21]. However, the cellular source of these transcripts, the localization of testicular PDGF-C protein, its proteolytic processing, and the physiological function of PDGF-C in the adult testis were not determined. These ligands activate PDGFRα and PDGFRβ as homo- or heterodimeric receptor tyrosine kinases, with ligand–receptor selectivity and receptor abundance determining the downstream response [22,23,24]. Receptor phosphorylation recruits signaling modules, including phosphoinositide 3-kinase (PI3K)–AKT, RAS–MAPK, phospholipase Cγ, and SRC-family pathways [25,26]. The resulting outputs, such as survival, proliferation, migration, differentiation, and matrix production, are strongly dependent on cell identity and tissue context [22,27].

The clearest evidence of reproduction concerns PDGF-A–PDGFRα signaling during testis development. Sertoli-cell-derived PDGF-A acts on PDGFRα-positive interstitial progenitors, contributing to fetal Leydigcell differentiation and testis cord organization [15,16]. Disruption of *Pdgfa* results in progressive Leydigcell loss, reduced testis size, and spermatogenic arrest, while *Pdgfra* deficiency disrupts testicular morphogenesis and the Leydig lineage [15,16,17]. These findings establish an important boundary condition for any therapeutic proposal: PDGFRα cannot be treated simply as a pathological receptor whose inhibition is expected to improve fertility.

Recent mechanistic work resolves how this developmental function is executed. In *Pdgfra*-null fetal XY gonads, ERK activity was markedly reduced, testis cords were fewer and abnormally organized, and terminal steroidogenic markers, including Cyp11a1, Hsd3b1, and Cyp17a1, were decreased despite preservation of a broad NR5A1-positive interstitial population [28]. Ex vivo, cellular and genetic experiments showed that PDGFRα-dependent ERK signaling promotes testis cord formation through EGR1-mediated cell migration. In contrast, ERK-dependent CREB activation supports the terminal fetal Leydigcell steroidogenic program. PDGFRα, therefore, regulates both tissue architecture and functional differentiation rather than simply determining Leydig-cell abundance.

PDGF signaling also remains relevant after fetal development. PDGFRα expression has been detected in adult Leydig cells and peritubular or interstitial cell populations [14,29]. In stem Leydig cells, changes in PDGFRα and PDGFRβ expression accompany differentiation, and PDGF isoforms differentially regulate proliferation and lineage progression [17]. These observations indicate that receptor isoforms should not be considered interchangeable. PDGFRα is most strongly linked to Leydig-lineage development and progenitor-associated regulation, whereas PDGFRβ is more commonly associated with vascular mural cells, pericytes, and activated mesenchymal populations. However, the adult testicular distribution of both receptors and their age- or injury-related changes require systematic mapping.

Direct evidence for this expression pattern in humans comes from an immunohistochemical mapping study of PDGF-A, PDGF-B, and PDGFRα/β across fetal and adult human testis, which localized PDGFRα predominantly to Leydigcells and peritubular tissue and additionally reported altered receptor expression patterns in testicular disease states, including Leydig-cell tumors and Sertoli-cell-only pattern testes [30]. The Leydig-cell-selective expression of PDGFRA is at least partly explained at the transcriptional level. In the TM3 Leydigcell line, *Pdgfra* promoter activity depends on the transcription factors specificity protein 1 (Sp1) and Sp3 acting on a GC-rich proximal promoter element, offering one mechanism by which PDGFRA expression could be preferentially retained in steroidogenic-lineage cells relative to neighboring stromal populations [31]. A second independent transcriptional input converges on the same gene: the forkhead transcription factor FOXA3, expressed in Sertoli cells throughout development and in Leydig cells postnatally, was shown to regulate *Pdgfra* transcription in Leydig cells directly, and Foxa3-deficient mice display progressive subfertility with germ-cell loss [32]. The existence of at least two distinct transcription-factor inputs (Sp1/Sp3 and FOXA3) suggests that *Pdgfra* expression is not fixed but is tunable, raising the possibility that its transcriptional control, rather than only its ligand-driven activation, could be a point of vulnerability to oxidative or endocrine disruption.

The physiological function of this pathway is also likely to depend on intercellular communication. Sertoli cells can provide PDGF ligands to neighboring interstitial cells; Leydig-derived androgens support the seminiferous epithelium; peritubular cells influence tubular structure and paracrine signaling; and vascular cells regulate oxygen and nutrient delivery. Consequently, altered PDGF–PDGFR signaling in one compartment may indirectly affect spermatogenesis via a cascade of niche-level changes rather than directly acting on germ cells.

### 3.2. Adult Testicular Stromal Populations

The meaning of PDGFRα expression changes with developmental stage and species. In rodents, PDGFRα is widely used to enrich stem or progenitor Leydig-cell populations, and PDGF ligands influence their proliferation and differentiation [14,17]. In the human testis, however, PDGFRα-positive interstitial cells display mesenchymal multipotency but do not necessarily acquire full testosterone-producing capacity after Leydig-directed differentiation [33]. PDGFRα should therefore be viewed as a marker of a stromal/progenitor-enriched compartment rather than as a species-independent identifier of a functional stem Leydig cell.

Compared with its well-established requirement during fetal testis development, the physiological function of PDGFRα signaling in the adult testis remains poorly defined. Available adult-stage evidence has primarily addressed receptor localization and transcriptional regulation. FOXA3 was detected in adult mouse Leydig cells and shown to regulate the *Pdgfra* promoter [32], whereas PDGFRα-associated reporter fluorescence and PDGFRα immunostaining were observed in the interstitial and peritubular compartments of 12-week-old mouse testes [29]. These findings establish adult expression, transcriptional regulation, and signaling capacity but do not demonstrate an in vivo requirement for PDGFRα in adult Leydig-cell maintenance, steroidogenesis, spermatogenic support, fertility, or regeneration after injury.

Human anatomical studies further reveal that PDGFRα expression is not confined to the Leydig lineage. CD34-positive/PDGFRα-positive telocytes form networks in the outer peritubular and intertubular stroma, surround Leydig cells and vessels, and establish close contacts with neighboring telocytes, peritubular myoid cells, and vascular structures [34]. These telocytes are negative for α-smooth-muscle actin and are therefore distinguishable from the myoid cells in the inner peritubular layer. Their processes and associated extracellular vesicles suggest a potential role in long-range stromal communication, although direct functional evidence remains limited.

PDGFRα positivity does not define a single testicular stromal lineage or imply a shared biological function. Depending on developmental stage, species, anatomical compartment, and detection method, PDGFRα-associated populations may include Leydig-lineage progenitors, TCF21-positive or CD34-positive mesenchymal cells, telocytes, and cells located within or adjacent to peritubular and perivascular compartments. These populations differ in lineage history, spatial location, differentiation capacity, and potential contributions to steroidogenesis, extracellular-matrix regulation, vascular support, and tissue repair. Moreover, reporter activity, transcript detection, and endogenous PDGFRα protein localization represent distinct measurements and should not be treated as interchangeable evidence. Findings obtained in one PDGFRα-positive population, therefore, cannot be generalized to other stromal compartments without cell-specific protein validation and functional perturbation. Similarly, bulk changes in *Pdgfra* transcript abundance or total PDGFRα protein cannot determine whether they reflect loss of a supportive population, expansion of a reparative progenitor compartment, altered expression within existing cells, or activation of matrix-producing stromal cells.

Our previous studies provide anatomical and indirect functional evidence relevant to this cellular distribution. In heterozygous PDGFRα-EGFP reporter mice, reporter-positive cells were observed predominantly in the testicular interstitium and peritubular compartment, with reported co-localization of PDGFRα-associated signals with c-KIT, ANO1, and TASK-1 [29]. Separately, pharmacological inhibition of c-KIT or ANO1 attenuated LH-induced intracellular Ca^2+^ elevation, membrane depolarization, and testosterone secretion in mouse Leydig cells [35]. These findings support the presence of these signaling components in anatomically related adult testicular populations but do not establish functional coupling among PDGFRα, c-KIT, ANO1, and TASK-1. Direct receptor activation and cell-specific perturbation will be required to test such interactions.

Cell-based evidence further indicates that PDGF-AA signaling can be spatially organized in testicular interstitial Leydig cells. PDGFRα was localized to primary cilia, and PDGF-AA stimulation activated AKT and ERK around the ciliary base and promoted cell growth, migration, and invasion [36]. Depletion of IFT88 or CEP164 attenuated PDGF-AA-induced AKT and ERK activation and reduced these cellular responses. These findings identify primary cilia as candidate PDGFRα signaling microdomains in the studied Leydig-cell model but do not establish their requirement for adult testicular homeostasis or reproductive function in vivo.

### 3.3. Postnatal Development and Iatrogenic Risk

The PDGFR axis is sensitive to exogenous perturbation during prenatal development. In rats, maternal exposure to diethylstilbestrol, bisphenol A, genistein, or coumestrol from gestational day 14 to birth generally increased whole-testis *Pdgfra* and *Pdgfrb* mRNA abundance in neonatal offspring in a dose-dependent manner, although diethylstilbestrol produced a biphasic response [37]. Importantly, these changes were not confined to gonocytes. In situ hybridization localized the increase in *Pdgfra* mRNA predominantly to the interstitium, whereas *Pdgfrb* mRNA increased in both the interstitium and seminiferous cords. Both receptors were detected in purified gonocytes, and PDGFRβ protein showed a particularly marked increase in gonocytes following estrogenic exposure [37]. These findings identify the developing PDGFR system as a target of estrogenic perturbation but do not establish increased receptor activation or a uniform response across testicular cell populations.

A more environmentally realistic combined-exposure study reinforces this concern. Simultaneous gestational exposure to the phytoestrogen genistein and the antiandrogenic plasticizer di-(2-ethylhexyl) phthalate (DEHP), a combination chosen to model real-world co-exposure rather than a single compound, produced long-term disruption of adult testis development and function in rat offspring, with altered expression of the Leydigcell-associated genes *Hsd3b*, *Anxa1*, *Foxa3*, and *Pdgfra* identified by gene-expression array and selected changes further validated by quantitative PCR and immunohistochemistry [38]. *Pdgfra* recurs as an expose-responsive gene across single-compound and mixture exposures and is also linked to transcriptional regulatory pathways involving Sp1/Sp3 and FOXA3, as described above. The recurrence of altered *Pdgfra* expression across these exposure models supports its consideration as a candidate marker of developmental endocrine disruption. However, its mechanistic and predictive significance remains to be established.

Postnatal testicular development depends on coordinated communication among germ cells, Sertoli cells, Leydig cells, and mesenchymal myoid precursors. In immature rats, imatinib administration on postnatal days 5–7 delayed gonocyte migration and formation of the spermatogonial stem-cell pool, reduced the proliferation of type-A spermatogonia, increased germ-cell apoptosis, reduced the proliferation of mesenchymal myoid precursor cells, and shortened seminiferous cords [39]. In adulthood, the treated rats had lower absolute testicular weight. They reduced total seminiferous-tubule length, although tubular diameter, epithelial thickness, and epididymal sperm counts were comparable to those of controls. Serum LH, FSH, and inhibin B concentrations were elevated, whereas intratesticular testosterone was unchanged [39]. The authors interpreted the hormonal changes as evidence of compensatory endocrine regulation, but compensation was not directly demonstrated.

A companion study from the same group examined reproductive function after the same early postnatal exposure. Treated males remained fertile, and their fertility index, live-birth index, offspring survival, and offspring sex ratio were unaffected; however, they sired significantly smaller litters [40]. At adulthood, the treated males also had lower body weight and lower absolute weights of the testes and epididymides, whereas Leydig-cell morphology, the relative proportions of Leydig and interstitial tissue, intratesticular testosterone, and testicular mRNA levels of the SCF/KIT and PDGF/PDGFR ligand–receptor systems did not differ significantly from controls [40]. No overt abnormalities were detected in the measured outcomes of the male offspring.

Collectively, these findings indicate that brief exposure to imatinib during early postnatal development can produce long-lasting changes in testicular growth, gonadotropin regulation, and reproductive output that are not fully reflected by adult histology, sperm counts, or ligand–receptor transcript abundance. However, because imatinib inhibits PDGFRα, PDGFRβ, KIT, ABL-family kinases, and c-FMS, and because systemic growth was also affected, these studies do not establish that PDGFRα inhibition specifically caused the adult reproductive phenotype. The pharmacological limitations of this interpretation are discussed further in Section 9.1.

A recent scoping review similarly concluded that imatinib exposure is associated with testicular toxicity, impaired spermatogonial proliferation, altered steroidogenesis, and possible disruption of the blood–testis barrier, with neonatal or prepubertal exposure appearing particularly consequential [41]. These observations have clinical relevance for fertility counseling, but they should not be interpreted as proof that all gonadotoxicity is mediated by PDGFR inhibition. c-KIT is essential for germ-cell survival, while PDGFR signaling is more directly implicated in Leydig and mesenchymal compartments. Selective genetic or pharmacological separation of these targets remains necessary.

### 3.4. Corpus Cavernosum Homeostasis and Repair

Male reproductive health includes the vascular and smooth-muscle integrity required for erectile function. A single-cell transcriptomic atlas of the human corpus cavernosum provided initial evidence that PDGFRA is expressed in the cavernosal fibroblast compartment. Zhao et al. profiled 64,993 cells from three men with normal erectile function and five patients with organic erectile dysfunction and identified fibroblasts using LUM, COL1A1, and PDGFRA expression. Fibroblasts comprised a heterogeneous population with six transcriptionally distinct subclusters. They exhibited strong outgoing signaling activity within the predicted cell–cell communication network, suggesting that they contribute to cavernosal microenvironmental homeostasis rather than serving solely as extracellular matrix-producing cells [42]. This human transcriptomic framework was extended by subsequent studies examining fibroblast function and reparative potential. In mice, SLC1A3-positive cavernosal fibroblasts were shown to regulate penile erection, although this population was not defined by PDGFRα expression [43]. More recently, single-cell RNA sequencing, spatial transcriptomics, and multiplex immunofluorescence identified a PDGFRα-positive/Sca1-positive fibroblast population associated with vessel walls in the corpus cavernosum [44]. These cells exhibited angiogenesis-related and progenitor-like features and were depleted in diabetic and cavernous nerve injury models of erectile dysfunction. Their depletion coincided with reductions in endothelial and smooth-muscle cell populations, establishing an association with impaired vascular maintenance but not demonstrating a causal role in repair.

Collectively, these studies broaden the biological interpretation of cavernosal fibroblasts and PDGFRα beyond fibrosis. However, the evidence must be interpreted at distinct levels. The human atlas establishes PDGFRA as a marker of the broader fibroblast compartment but does not demonstrate a receptor-specific function. The SLC1A3-positive population regulates erection in mice but has not been shown to correspond directly to PDGFRα-positive fibroblasts. The PDGFRα-positive/Sca1-positive population is associated with vascular repair, but computational differentiation trajectories and marker co-localization do not establish direct conversion to endothelial or smoothmuscle cells. Lineage tracing and selective depletion or restoration are therefore required to distinguish direct progenitor activity from paracrine support. This distinction is particularly important under oxidative conditions, such as those seen in diabetes, in which failed stromal repair and excessive matrix deposition may coexist.

**Figure 2:**
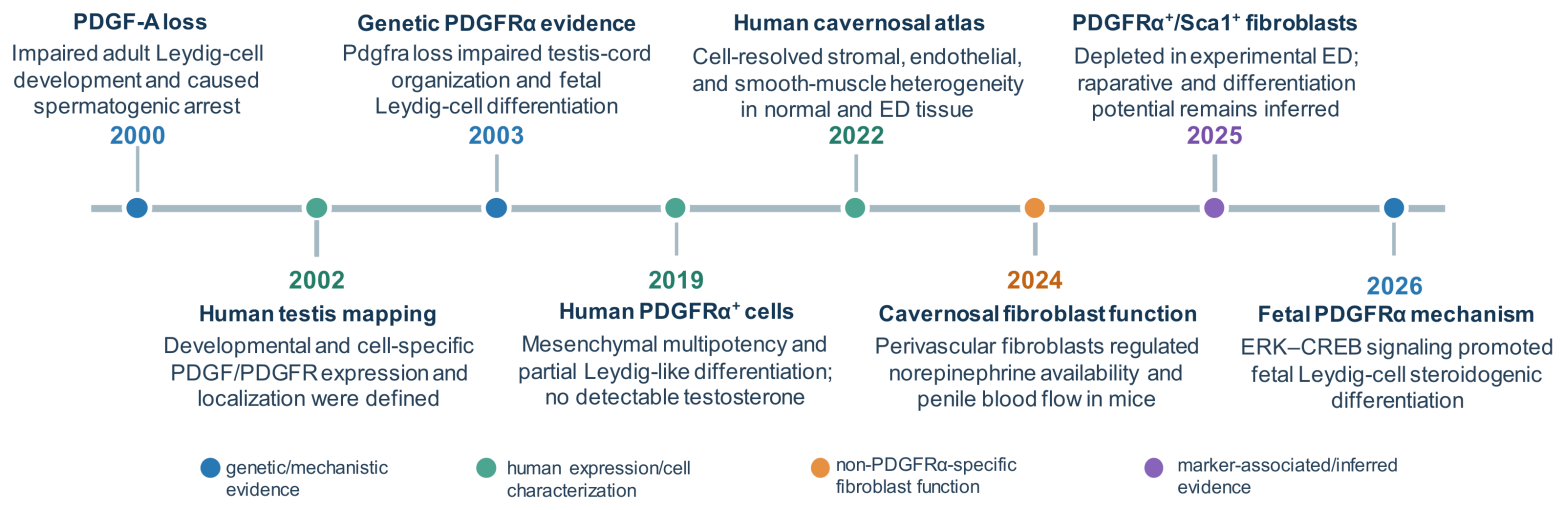
Selected milestones in PDGFRα research in male reproductive biology. The timeline summarizes the progression from developmental expression mapping and mouse genetic studies to cell-resolved characterization of testicular and cavernosal stromal populations [15,16,28,30,33,42,43,44]. Evidence ranges from direct genetic and mechanistic studies of fetal testis development to human descriptive datasets and preclinical studies identifying candidate adult stromal and reparative functions. The cavernosal fibroblast study in mice [43] is included as contextual evidence because the functionally characterized cells were not established as a PDGFRα-positive population. Proposed lineage or reparative roles require further cell-specific causal validation.

Taken together, the studies discussed in Section 3.1, Section 3.2, Section 3.3 and Section 3.4 illustrate the progression of PDGFRα research from developmental expression mapping and genetic analysis to cell-resolved characterization of adult testicular and cavernosal stromal populations. Figure 2 summarizes selected milestones in this progression across different species, developmental stages, and experimental models.

## 4. Reciprocal Coupling Between PDGFR Signaling and Redox State

Growth-factor signaling is not merely affected by reactive oxygen species (ROS); it can also generate ROS as second messengers. In non-reproductive cell models, PDGF stimulation induces Rac-dependent NADPH oxidase activity and localized ROS production [18,19,20]. PDGF-AA-induced ROS generation has been linked to PI3K-dependent recruitment of Rac1 and p47phox to the plasma membrane [18]. This finding supports the involvement of a dynamically assembled, Rac-responsive NOX complex. Still, it does not identify the catalytic NOX isoform. It should not be generalized to NOX4, which does not require Rac1- or p47phox-dependent complex assembly.

Hydrogen peroxide can reversibly oxidize the catalytic cysteine of protein tyrosine phosphatases (PTPs), thereby transiently reducing their activity. PDGF-induced oxidation of the PDGFR-associated phosphatase SHP-2 has been demonstrated in fibroblasts, providing a mechanism through which ROS may prolong receptor-associated tyrosine phosphorylation [45,46]. PDGFR activation and PTP oxidation could therefore form a bidirectional circuit in which receptor stimulation generates ROS, which in turn reinforces signaling by limiting dephosphorylation. However, PTP oxidation is not specific to PDGFRα and may also affect KIT, EGFR-family receptors, insulin and IGF receptors, FGFRs, VEGFRs, and other receptor tyrosine kinases. Similarly, activation of shared downstream effectors such as AKT and ERK cannot establish PDGFRα-specific signaling.

Whether such coupling occurs in Leydig-lineage cells, Sertoli cells, peritubular cells, or other testicular interstitial cells has not been demonstrated. The relevant evidence is derived predominantly from vascular smooth-muscle cells, fibroblasts, lens epithelial cells, and other non-reproductive models [18,19,20,45,46]. The catalytic NOX isoform, its dependence on Rac1, and the relationship between NOX-derived ROS and PDGFRα phosphorylation remain unknown in male reproductive tissues. Accordingly, the proposed transient-versus-persistent model does not assume that the two states are mediated either by different temporal behaviors of a single NOX isoform or by distinct NOX isoforms. Direct validation will require cell-resolved mapping of PDGFR and NOX isoforms, simultaneous measurement of localized ROS and receptor phosphorylation, and receptor- or NOX-specific perturbation.

PDGFR-associated redox signaling should also be evaluated alongside established testicular stress-response pathways. The NRF2–KEAP1 pathway regulates antioxidant capacity, and genetic loss of *Nrf2* causes age-dependent oxidative injury, germ-cell apoptosis, and impaired spermatogenesis in mice [47]. NF-κB and stress-activated MAPKs connect oxidative and inflammatory stimuli to cytokine production and testicular injury [4]. TGF-β3–p38 MAPK signaling contributes to blood–testis-barrier remodeling [48], whereas TGF-β/SMAD signaling has been implicated in fibroblast-mediated extracellular-matrix deposition and fibrosis in cadmium-exposed testis [49]. PDGFRα is therefore proposed neither as an alternative to these pathways nor as a dominant regulator of testicular oxidative stress, but as a candidate cell-surface signaling system that may intersect with them in defined stromal populations. Such interactions should not be inferred from changes in shared downstream mediators alone but require cell-resolved assessment of PDGFRα activation and pathway-specific perturbation.

## 5. Peritubular Myoid Cells as a Candidate Redox-PDGFR Interface

Peritubular myoid cells form one or more cellular layers around the seminiferous tubules and contribute to tubular architecture, contractility, extracellular-matrix organization, and paracrine communication [50,51]. Their contractions assist the movement of luminal fluid and non-motile testicular sperm toward the rete testis. Because these cells lie at the boundary between the seminiferous epithelium and the interstitial compartment, their dysfunction could translate an interstitial oxidative signal into both mechanical and biochemical impairment of spermatogenesis.

A potentially important but incompletely tested possibility is that persistent oxidative and growth factor signaling shifts peritubular myoid cells from a predominantly contractile state toward an extracellularmatrix-producing state. Functionally analogous transitions occur in vascular smoothmuscle cells and fibrosis-responsive mesenchymal cells in other organs [52,53], but peritubular myoid cells should not be regarded as direct equivalents of hepatic stellate cells or other canonical myofibroblast precursors. Moreover, evidence that PDGFR signaling itself drives this transition in the testis remains limited. The appropriate formulation is therefore a testable model: redox-dependent prolongation of PDGFR activity may contribute to peritubular remodeling, rather than an established causal pathway.

This model predicts two interacting consequences. First, reduced or uncoordinated contractility may impair tubular fluid transport. Second, excessive deposition of collagen, fibronectin, or other matrix components may thicken the peritubular compartment and alter the diffusion of oxygen, nutrients, androgens, and paracrine factors between interstitial and seminiferous cells. Together, these changes could compromise Sertoli-cell support and germcell development, even in the absence of a direct effect of PDGFR signaling on spermatozoa. Phenotype should be evaluated using a marker panel rather than α-smooth-muscle actin alone: MYH11, CNN1, TAGLN, and DES can inform contractile identity, whereas COL1A1, COL3A1, FN1, and POSTN can indicate matrix-producing activation. ACTA2/α-smooth-muscle actin requires context-dependent interpretation because it is associated with both contractility and myofibroblastic activation [54,55].

Receptor assignment also requires caution. PDGFRβ is frequently associated with vascular mural cells and pericytes [56], but it should not be assumed to be a universal marker of all peritubular myoid cells. Spatial mapping of PDGFRα and PDGFRβ across the inner and outer peritubular layers, interstitial mesenchyme, and testicular vasculature is therefore required before receptor-specific functions can be assigned to these populations. More broadly, the evidence underlying this framework spans different species, developmental stages, tissues, and experimental approaches. To clarify the directness and relevance of this evidence to reproductive tissue, Table 1 summarizes the representative studies central to this review, including their experimental models, study designs, principal findings, evidence categories, and key limitations. These categories are intended to facilitate interpretation and do not constitute a formal risk-of-bias or methodological-quality assessment.

## 6. A Context-Dependent Model

As a working hypothesis, PDGFR-associated responses during oxidative testicular stress may separate into adaptive support and maladaptive remodeling. “Transient signaling” refers, operationally, to a localized and reversible ROS and phosphorylation response that returns toward baseline after removal of the stimulus, without sustained cellular dysfunction or matrix induction. “Severe oxidative injury” refers to an oxidant burden that exceeds cellular antioxidant and repair capacity, resulting in macromolecular damage, loss of differentiated function, or reduced cell viability. “Persistent oxidative or inflammatory stress” refers to sustained or recurrent ROS and inflammatory signaling that fails to resolve and is accompanied by prolonged cellular dysfunction or matrix-gene induction across serial time points. These operational categories are distinguished by the magnitude, duration, localization, reversibility, and biological consequences of the response rather than by fixed ROS concentration thresholds. They are proposed experimental categories, not validated clinical states.

Within this framework, transient PDGFR-associated signaling may support cell survival, differentiated function, or tissue repair. A distinct but unproven possibility is that unresolved injury promotes a remodeling response in peritubular, vascular, or interstitial mesenchymal populations. Persistent growth factor signaling could promote migration, proliferation, and extracellular matrix synthesis, but PDGFRα-specific causality has not been established in the testis. The hypothesis should therefore be tested against TGF-β, inflammatory, mechanical, and other receptor tyrosine kinase pathways rather than treating fibrosis as a PDGFRα-mediated endpoint.

These two potential roles are not necessarily contradictory. The same ligand–receptor family may preserve one cell population while promoting pathological activation in another. Outcome is likely to depend on four variables: (i) receptor isoform and dimer composition; (ii) responding cell type; (iii) ROS intensity, localization, and duration; and (iv) stage of injury. Early receptor activation may support survival and repair, whereas persistent activation after unresolved injury may favor fibrosis. Alternatively, severe oxidative stress may impair PDGFRα-positive Leydig-lineage cells or their signaling while concurrently promoting stromal activation through inflammatory mediators and multiple growth-factor pathways. This potential cell-specific divergence provides a more coherent model than classifying PDGFR signaling as uniformly beneficial or harmful. Table 2 integrates the established reproductive-tissue findings and proposed context-dependent relationships by cellular compartment, reproductive implication, and evidence base.

## 7. Implications for Male Reproductive Aging and Disease

Aging, obesity, and diabetes combine mitochondrial oxidative stress with chronic low-grade inflammation, endocrine disturbance, and vascular dysfunction [3,4,13,61]. These conditions could alter PDGF ligand production, receptor expression, and the balance between Leydigcell maintenance and interstitial remodeling. In the aging mouse testis, Leydig cells appear particularly susceptible to senescence, as supported by interstitial senescence-associated β-galactosidase activity, increased Leydig-cell p21, and cell-resolved transcriptomic changes [62]. Whether PDGFRα-positive mesenchymal or other interstitial stromal populations undergo similar age-associated changes remains unknown. Age-associated Leydig-cell dysfunction has been linked to oxidative stress, chronic inflammation, mitochondrial dysfunction, and impaired steroidogenesis [62,63,64]. Age-dependent single-cell and spatial studies are needed because bulk testicular measurements may mask opposing changes across distinct cellular compartments [63,65].

A further candidate link between aging and PDGFR-associated remodeling is cellular senescence. Senescent cells accumulate with age in many tissues and adopt a senescence-associated secretory phenotype (SASP) that includes growth factors, matrix-remodeling enzymes, and pro-inflammatory cytokines, several of which are known to engage PDGFR signaling in paracrine target cells in other organs. Whether senescent Leydig, peritubular, or interstitial cells accumulate in the aging testis, and whether their secretory output sustains PDGFR activation in neighboring stromal populations, has not been directly tested but represents a tractable extension of the framework proposed here [66]. If confirmed, senolytic or SASP-modulating strategies could provide an additional entry point to interrupt age-associated stromal remodeling, complementary to direct PDGFR-targeted approaches.

Oxidative stress and chronic low-grade inflammation frequently coexist in male reproductive dysfunction [3,4]. Whether PDGFRα directly regulates the testicular immune microenvironment remains unresolved. A study combining single-cell transcriptomics and lineage tracing identified a TCF21-positive mesenchymal population expressing *Pdgfra* that was molecularly distinct from testicular macrophages and contributed to adult testicular homeostasis and regeneration after injury [58]. Separately, transplantation of CD51-positive stem Leydig cells suppressed macrophage accumulation and inflammatory cytokine expression after testicular ischemia–reperfusion injury through TRPM7-dependent mitochondrial transfer [67]. However, that study did not examine whether PDGFRα expression or activation mediated these effects. Thus, current evidence demonstrates communication between testicular stromal/progenitor populations and macrophages but does not establish that PDGFRα is functionally expressed in testicular immune cells or that PDGFRα signaling mediates the local immune response. Cell-resolved receptor mapping and lineage-specific perturbation will be required to test this possibility.

Environmental toxicants, heat, and ionizing radiation can induce ROS generation, DNA damage, mitochondrial dysfunction, apoptosis, and inflammatory signaling in testicular cells [68,69,70]. In a previous mouse study, chronic low-dose-rate radiation increased testicular *Pdgfra* mRNA expression, particularly at the highest dose rate, together with collagen deposition and other fibrotic changes [59]. However, because *Pdgfra* was measured in bulk testicular tissue without assessing receptor phosphorylation or cellular origin, the biological significance of this increase remains unresolved. It could reflect compensatory repair, expansion of PDGFRα-positive stromal populations, maladaptive remodeling, or divergent responses among distinct cell populations. Bulk *Pdgfra* mRNA abundance alone cannot discriminate among these possibilities. Chronic low-dose-rate radiation or phthalate exposure should not be assumed to activate a testicular PDGFR–NOX circuit unless receptor phosphorylation, ligand availability, ROS source, and cell identity are demonstrated directly. Time-resolved, cell-resolved analysis is therefore more informative than a single terminal measurement of bulk testicular *Pdgfra* mRNA abundance.

A single-cell RNA-sequencing atlas of testes from BPA-exposed mice further illustrates the cell-pair specificity of this response. Although the predicted overall number and strength of intercellular interactions increased following BPA exposure, ligand–receptor analysis identified reduced *Pdgfa–Pdgfra* communication involving Leydig cells and pachytene spermatocytes [57]. Because this finding was inferred from single-cell transcriptomic data, it does not directly demonstrate reduced PDGFRα protein abundance, receptor phosphorylation, or downstream signaling activity. This finding should not be interpreted as directly contradicting the receptor expression changes reported by Thuillier et al. [37]. The studies differed in species, exposure window, developmental stage, cellular populations, and analytical endpoint: Thuillier et al. measured receptor mRNA and protein abundance in neonatal rat testis following prenatal exposure [37], whereas Xia et al. inferred the strength of communication between defined cell populations from single-cell transcriptomic data [57]. Moreover, an inferred ligand–receptor interaction score does not directly measure PDGFRα phosphorylation or downstream signaling activity. Collectively, these studies indicate that endocrine-disrupting exposure can alter the testicular PDGFR system in a context- and cell-type-dependent manner. Still, they do not establish whether BPA produces a uniform increase or decrease in overall PDGFR signaling.

Complementary single-cell evidence comes from cadmium exposure, in which Sertoli-cell-derived extracellular vesicles promoted testicular inflammation and fibrosis, accompanied by Sertolicell loss and fibroblast expansion [49]. Together, these studies illustrate how toxicants can alter intercellular and stromal programs in a cell-type-specific manner without necessarily activating a generic PDGFR–NOX circuit.

Cryopreservation and prolonged incubation primarily expose spermatozoa to oxidative injury and are unlikely to involve an intact testicular niche [71,72,73]. PDGFR-directed concepts should therefore not be extended uncritically to all forms of male reproductive oxidative stress. Their greatest relevance is expected in vivo, where somatic-cell communication and tissue remodeling influence sperm production before ejaculation. This distinction preserves the scope of the hypothesis and avoids conflating testicular pathophysiology with oxidative damage arising during semen handling.

## 8. Prostate and Accessory Reproductive Organs

The prostate is part of the male reproductive system, but the PDGF–PDGFR literature in this organ is dominated by cancer biology rather than normal reproductive physiology. Much of the strongest evidence concerns PDGFRβ-positive tumor-associated stroma, PDGF-B or PDGF-D signaling, macrophage–stromal crosstalk, and associations with recurrence [74,75]. These findings demonstrate the importance of PDGFR signaling in prostate stromal plasticity but do not define the physiological role of PDGFRα-positive cells in the normal gland.

For this reason, prostate cancer should not dominate a review focused on oxidative stress and male reproductive function. Instead, it exposes a broader knowledge gap: the identities and homeostatic functions of PDGFRα-positive stromal cells remain poorly resolved in the normal prostate, epididymis, vas deferens, and seminal vesicle. Future single-cell, spatial, and lineage-resolved studies should determine whether these accessory organs contain reparative PDGFRα-positive populations analogous to those identified in the testis and corpus cavernosum, and whether oxidative or inflammatory stress redirects them toward fibrosis.

The epididymis is a particularly relevant gap because it is the site of post-testicular sperm maturation. This process is itself redox-dependent: controlled oxidation of protamine cysteine residues and other epididymal maturation events rely on a tightly regulated luminal redox environment. Epididymal oxidative stress is an established contributor to defective sperm function independent of testicular pathology [4,8]. The epididymal stroma contains PDGFR-expressing peritubular and interstitial populations analogous to those described in the testis, but their contributions to maintaining this redox microenvironment and their responses to oxidative or inflammatory insults have not been systematically characterized. Given that epididymal dysfunction can produce a fertility phenotype that mimics testicular oxidative injury on standard semen analysis, resolving whether PDGFR-positive epididymal stromal cells constitute a comparable homeostatic network is a logical extension of the questions raised for the testis and corpus cavernosum.

## 9. Therapeutic Consideration for Context-Dependent PDGFRα Signaling

The proposed framework does not justify indiscriminate systemic inhibition of PDGFR. Such treatment could suppress pathological stromal activation but also interfere with vascular homeostasis, Leydig-lineage maintenance, or tissue repair [76]. These potential effects are context-dependent; however, direct evidence demonstrating the adverse consequences of systemic PDGFRα inhibition in the adult testis remains limited. Likewise, nonspecific antioxidant therapy could reduce damaging oxidants while attenuating the physiological ROS pulses required for receptor signaling and sperm function [9,10,11,12]. The two interventions, therefore, present related but mechanistically distinct risks: excessive PDGFR inhibition may produce deficient trophic and reparative signaling, whereas excessive antioxidant exposure may disrupt physiological redox signaling and, under some conditions, contribute to reductive stress.

This therapeutic caution is consistent with evidence from systematic reviews and current professional guidance on antioxidant supplementation in male infertility. The 2022 Cochrane review found that antioxidant supplementation may improve live-birth and clinical-pregnancy rates in subfertile men. However, the evidence was rated as very low certainty for live birth and low certainty for clinical pregnancy. Moreover, the apparent live-birth benefit was no longer evident after studies at high risk of bias were excluded. The review, therefore, concluded that the overall evidence remained inconclusive because of a serious risk of bias, incomplete reporting of clinically relevant outcomes, imprecision, and generally small study populations [5]. Professional guidelines similarly caution against routine empirical supplementation. The AUA/ASRM guideline states that the clinical utility of supplements, including antioxidants and vitamins, is questionable and that existing data are inadequate to recommend specific agents [6]. Similarly, the EAU guideline concludes that there are no conclusive data supporting antioxidant treatment in men with idiopathic infertility and provides a weak recommendation against its routine use in this population [7]. These conclusions do not demonstrate that antioxidant therapy is uniformly ineffective or harmful. Rather, they argue against routine empirical supplementation in unselected patients and support the need for better patient stratification according to the underlying cause and relevant redox pathology. From the perspective proposed here, the therapeutic objective should not be maximal suppression of ROS or PDGFRα signaling, but context-appropriate modulation of their spatial and temporal signaling patterns.

This duality is consistent with our previous organ-level review of PDGF/PDGFR signaling, which emphasized that the same pathway supports physiological development, growth, and tissue regeneration but promotes fibrosis, vascular remodeling, and cancer when activation becomes excessive or persistent [22]. In male reproductive tissues, therapeutic design should therefore be guided by cellular source, receptor isoform, activation state, and disease stage rather than by total PDGFRα abundance alone.

A more appropriate therapeutic objective would be to maintain or restore PDGFR signaling within a physiological spatial and temporal range. The proposed framework suggests three context-dependent objectives: preserving transient signaling required for steroidogenesis, stromal homeostasis, and vascular repair; restoring supportive PDGFRα-positive populations after severe injury; and selectively restraining persistent matrix-producing or profibrotic signaling. Potential strategies include receptor isoform-selective modulation, delivery to defined reproductive cell populations, interruption of pathological ligand–receptor interactions, or inhibition of downstream profibrotic pathways while preserving survival and repair signaling. These approaches remain hypothetical until receptor distribution and causal function are resolved in relevant disease models.

Cell-resolved PDGFR status, defined by receptor abundance, spatial distribution, and phosphorylation state, may therefore initially be more useful as a mechanistic biomarker of stromal state and pathway activity than as a direct therapeutic target. Combining cell-resolved PDGFRα or PDGFRβ abundance and receptor phosphorylation with oxidative damage, steroidogenic, and extracellular matrix markers may help distinguish diminished Leydig-lineage support from activation of matrix-producing stromal populations [22,36,60,77,78,79]. Bulk measurement of total PDGFR abundance alone cannot distinguish these biologically distinct states.

This context-dependent therapeutic framework requires pharmacological approaches that attenuate sustained pathological signaling while preserving transient physiological PDGFRα activity. However, currently available tyrosine kinase inhibitors do not provide this degree of cellular or signaling selectivity.

### 9.1. Limitations of Current Pharmacological Inhibition

Several small-molecule tyrosine kinase inhibitors, including imatinib, sunitinib, nintedanib, and crenolanib, inhibit PDGFRα, but none is selective exclusively for this receptor. Imatinib inhibits PDGFRα and PDGFRβ together with KIT and ABL-family kinases. Sunitinib inhibits both PDGFR isoforms as well as VEGFRs, KIT, FLT3, and several other receptor tyrosine kinases, whereas nintedanib primarily targets PDGFRα/β, VEGFR1–3, and FGFR1–3 [80]. Crenolanib has potent activity against PDGFRα but also inhibits PDGFRβ and FLT3 [81]. Moreover, the degree of target inhibition depends on drug concentration, exposure duration, and cellular context. Consequently, biological effects observed after treatment with these compounds cannot be attributed specifically to PDGFRα inhibition without complementary genetic, target-engagement, or rescue experiments.

This lack of selectivity is particularly consequential in the male reproductive system. KIT signaling is important for germ-cell development and survival, VEGF signaling contributes to testicular vascular maintenance, and physiological PDGF–PDGFRα signaling participates in testis cord organization and the development of Leydig-lineage and interstitial populations [15,22,31,33,36]. Simultaneous inhibition of these pathways may therefore disrupt testicular physiology independently of any intended effect on pathological PDGFRα signaling. The early postnatal imatinib studies discussed in Section 3.3 illustrate this interpretive limitation [39,40]. Although the authors related the germ-cell and myoid-cell findings to concurrent disruption of KIT- and PDGFR-dependent developmental pathways, imatinib also inhibits PDGFRβ, ABL-family kinases, and c-FMS. Moreover, treated animals exhibited persistent reductions in body size and in absolute reproductive organ weights. The adult reproductive findings, therefore, cannot be specifically attributed to PDGFRα inhibition. Broader experimental and clinical literature has also associated imatinib and other multi-target tyrosine kinase inhibitors with alterations in testicular function and steroidogenesis, particularly following exposure during immature developmental stages [41,82,83]. However, the evidence is heterogeneous across drug, dose, exposure window, species, and outcome, and cannot be used to infer a class-wide or PDGFRα-specific reproductive effect.

Additional limitations include systemic exposure, dose-limiting adverse effects, variable tissue penetration, drug–drug interactions, and the potential emergence of kinase-domain mutations that reduce inhibitor sensitivity. The consequences of chronic PDGFRα inhibition for fertility and testicular endocrine function also remain insufficiently characterized because most clinical development programs have focused on cancer or fibrotic disease rather than reproductive outcomes. These limitations do not diminish the therapeutic value of existing tyrosine kinase inhibitors for established clinical indications, but they restrict their suitability for long-term preventive or fertility-preserving interventions.

These considerations provide a rationale for identifying alternative approaches that modulate rather than completely abolish PDGFRα signaling. Natural product-derived compounds may provide chemically diverse scaffolds that can influence receptor activation, downstream redox and kinase pathways, or cellular responses to excessive PDGF stimulation [84,85,86]. Their potential value should not, however, be inferred from their natural origin. Candidate compounds require rigorous evaluation of potency, direct target engagement, PDGFRα selectivity, kinase-wide off-target activity, pharmacokinetics, reproductive toxicity, and effects on the physiological functions of PDGFRα-expressing cells.

### 9.2. Future Perspective on Natural Product-Derived Scaffolds

As a future research direction, natural products may provide chemically diverse starting scaffolds for investigating context-dependent PDGFRα modulation. Still, their natural origin does not establish target selectivity, efficacy, or reproductive safety. Several natural-product-derived compounds have been reported to modulate PDGF-induced proliferation, migration, or fibrotic responses 85,87]. Curcumin was selected as a worked example because studies in non-reproductive mesenchymal cells have associated it with suppression of PDGF-induced responses and reductions in PDGFR expression or phosphorylation [85,87,88]. Tetrahydrocurcumin was included as a mechanistically informative comparator because it is a reduced metabolite of curcumin that retains reported antioxidant and antifibrotic activities but has no established direct activity against PDGFRα [89,90,91]. This parent compound–metabolite pair was selected for hypothesis generation rather than because either compound has demonstrated superiority over other candidates.

Importantly, the available evidence derives from non-reproductive models and, for curcumin, predominantly concerns PDGFRβ-associated responses. Neither compound has been shown to bind selectively to PDGFRα, inhibit PDGF-AA-induced PDGFRα activation, or modulate PDGFRα signaling in male reproductive tissue. Direct target engagement and receptor phosphorylation studies in validated PDGFRα-positive reproductive cells would therefore be required before either compound could be considered a validated PDGFRα-directed modulator. Accordingly, curcumin and tetrahydrocurcumin are discussed solely as illustrative examples for future research rather than as validated PDGFRα inhibitors, translational candidates, or therapeutic recommendations for male reproductive disorders.

**Figure 3:**
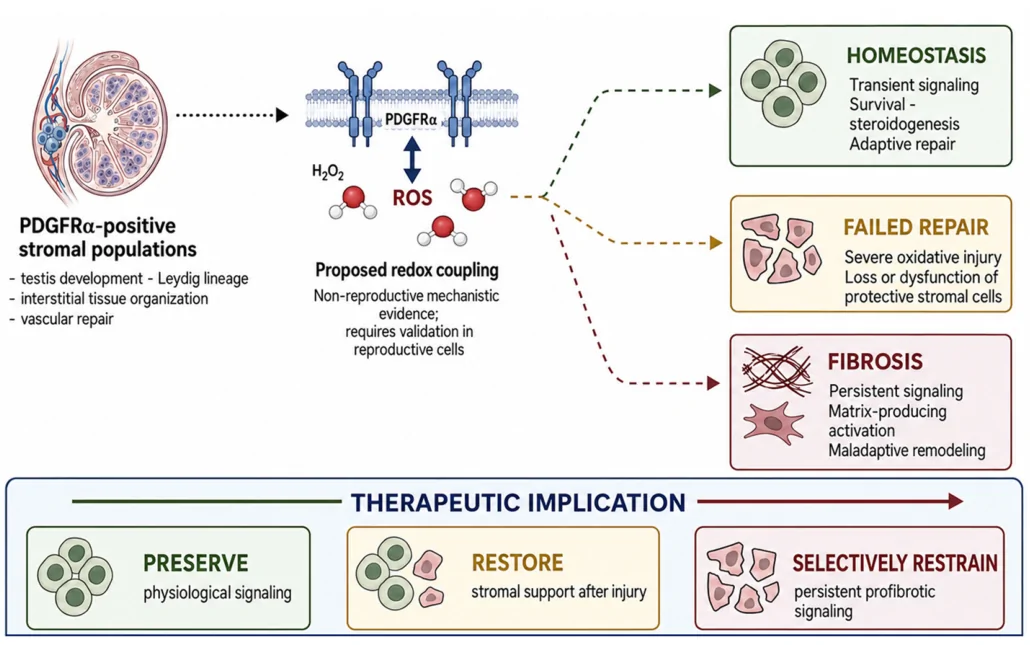
Proposed PDGFRα-associated stromal–redox framework and future research implications. The framework integrates reproductive tissue findings with redox mechanisms extrapolated primarily from non-reproductive systems and distinguishes adaptive signaling, injury-associated loss of stromal support, and persistent profibrotic remodeling. The corresponding objectives, preserving physiological signaling, restoring stromal support, and selectively restraining maladaptive signaling, are conceptual and have not been validated in male reproductive tissues. Dashed connectors indicate proposed relationships requiring direct validation.

Figure 3 summarizes the proposed framework linking PDGFRα-positive reproductive stromal populations and context-dependent redox outcomes to their potential therapeutic implications.

## 10. Research Agenda

Several questions must be answered before the PDGF–PDGFR axis can be assigned a causal role in oxidative male reproductive dysfunction.

1. **Map receptor and ligand expression at cellular resolution.** Single-cell and spatial transcriptomic datasets can identify candidate ligand-producing and receptor-expressing populations, but transcript detection does not establish protein abundance, membrane localization, or receptor activation. Transcriptomic findings should therefore be validated using cell-resolved protein methods, including multiplex immunofluorescence or immunohistochemistry with established lineage markers, RNA in situ hybridization combined with protein detection, and, where feasible, imaging mass cytometry or other spatial proteomic approaches. PDGFRα and PDGFRβ should be evaluated separately across Leydig-lineage, Sertoli, peritubular myoid, fibroblast, endothelial, pericyte, immune, and germ-cell populations. Cell identity should be assigned using combinations of lineage markers rather than a single marker, particularly because PDGFRα expression can span heterogeneous interstitial populations. PDGF-C represents a specific unresolved example: although *Pdgfc* transcripts have been detected in the adult mouse testis, the cell population that produces it, its protein localization, proteolytic activation, and reproductive function remain unknown. Cell-resolved localization and functional studies are needed to determine whether PDGF-C contributes to PDGFRα signaling in the adult testis.
2. **Distinguish receptor abundance from receptor activation.** Changes in *Pdgfra* or *PDGFRA* transcript abundance, total PDGFRα protein, and receptor phosphorylation represent different biological measurements and may not change in parallel. Receptor activation should be evaluated using validated phospho-PDGFRα assays together with measurements of downstream AKT, ERK, and PLCγ signaling. Whenever possible, these measurements should be performed in defined PDGFRα-positive cell populations using multiplex tissue imaging, phospho-flow cytometry, immunoprecipitation followed by immunoblotting, or cell-resolved phosphoproteomic approaches. Because receptor phosphorylation is transient and sensitive to tissue handling, sampling time, fixation conditions, and phosphatase inhibitor use, these should be standardized and reported. Time-course analysis after ligand stimulation or oxidative challenge is required to distinguish transient physiological activation from sustained or dysregulated signaling.
3. **Standardize validation and interpretation.** Antibody specificity should be confirmed using appropriate positive and negative controls, preferably including receptor-deficient tissue and an orthogonal detection method. Reporter expression should not be equated with endogenous receptor abundance or activation, and co-localization should not be interpreted as evidence of functional coupling. Dissociation-based methods should be complemented by in situ analysis because cell isolation removes spatial context and may alter phosphorylation states.
4. **Resolve ROS source, location, and kinetics.** Genetically encoded redox sensors and isoform-specific NOX perturbation should be used to distinguish localized signaling ROS from mitochondrial or inflammatory oxidative damage. Transcript and protein expression of NOX isoforms and their regulatory subunits should be mapped at cellular resolution across Leydig-lineage, Sertoli, peritubular, stromal, vascular, and immune populations. Isoform-specific genetic perturbation, combined with localized ROS measurements and time-resolved PDGFRα phosphorylation, will be required to determine whether stimulus-coupled and persistent ROS arise from the same NOX isoform or from distinct enzymatic sources. Species differences should also be considered by evaluating NOX1–NOX4 in rodent models and the corresponding NOX repertoire, including NOX5, in human tissues.
5. **Test bidirectionality.** Experiments should determine whether PDGFR activation generates ROS in testicular somatic cells and whether controlled oxidant exposure alters PDGFR phosphorylation via redox-sensitive phosphatases.
6. **Establish cell-specific causality in vivo.** Inducible lineage-restricted deletion or activation of *Pdgfra* and *Pdgfrb* is needed because pharmacological inhibitors cannot reliably separate receptor isoforms, cell populations, or developmental effects.
7. **Link molecular changes to reproductive outcomes.** Testosterone, testicular histology, and oxidative markers should be integrated with sperm count, motility, DNA integrity, mating success, and offspring outcomes.
8. **Define the therapeutic window.** Interventions should be tested at injury initiation, resolution, and established fibrosis to determine when PDGFR activity is reparative and when it becomes maladaptive.
9. **Evaluate candidate modulators without presuming target specificity.** Any natural-product-derived scaffold, including the illustrative curcumin–tetrahydrocurcumin pair discussed above, requires direct target-engagement, selectivity, reproductive safety, and cell-resolved signaling studies before it can be considered a PDGFRα-directed candidate.
10. **Translate findings into accessible human material.** Because rodent and human PDGFRα-positive interstitial populations differ in steroidogenic capacity, biobanked human testicular biopsy, orchiectomy, and corpus cavernosum tissue, together with human induced pluripotent stem cell-derived Leydig-like and peritubular-like cells, will be required to determine whether the mechanisms proposed here reproduce in human tissue before any translational application is considered.

## 11. Conclusions

Current evidence establishes an essential role for PDGFRα signaling in fetal testis development and identifies heterogeneous PDGFRα-positive stromal populations in adult reproductive tissues. Preclinical studies further suggest that selected PDGFRα-positive fibroblast populations in the corpus cavernosum have reparative functions. However, direct evidence linking PDGFRα signaling to redox regulation, immune responses, or pathological remodeling in the adult testis remains limited. In particular, it is unknown which adult testicular cell populations produce and respond to individual PDGF ligands, whether PDGFRα activation generates spatially restricted ROS through specific NOX isoforms, and whether oxidative stress suppresses reparative signaling or promotes persistent stromal activation.

Testing these hypotheses will require cell-resolved mapping of ligand and receptor proteins, validated measurements of receptor phosphorylation, genetically encoded redox sensors, and isoform-specific perturbation of NOX. Temporally controlled, lineage-restricted manipulation of *Pdgfra* and *Pdgfrb* in adult injury and aging models will be necessary to establish causality. These molecular measurements should be integrated with steroidogenesis, spermatogenesis, fibrosis, sperm quality, and fertility outcomes and subsequently validated in accessible human reproductive tissues or multicellular human models. Until such evidence is available, PDGFRα-associated redox signaling should be regarded as a testable mechanistic framework rather than an established therapeutic target.

## Figures and Tables

**Table 1:** Selected experimental studies relevant to PDGF–PDGFR signaling and PDGFRα-positive populations in male reproductive tissues.

Species and Developmental Stage	Tissue or Experimental Model	PDGF/PDGFR Assessment or Intervention	Principal Finding	Evidence Type and Key Limitation	Reference
Mouse, fetal	Developing XY gonads from *Pdgfra*-deficient embryos; gonad–mesonephros organ culture	Genetic disruption of *Pdgfra*: developmental, histological, proliferation, migration, vascular, and Leydig-cell analyses	*Pdgfra* loss impaired interstitial-cell proliferation, mesonephric cell migration, vascular organization, testis-cord formation, and fetal Leydig-cell differentiation	Direct in vivo developmental causality. Restricted to fetal development and does not establish adult-stage function	[15]
Mouse, postnatal to adult	*Pdgfa*-deficient testes	Genetic deletion of *Pdgfa*; testicular histology, hormone measurements, and developmental phenotyping	PDGF-A deficiency impaired development of the adult Leydig-cell population, leading to progressive Leydig-cell loss, undetectable testosterone in older mutants, and spermatogenic arrest	Direct in vivo ligand-level causality. Does not separate the contributions of different PDGFRα-containing receptor complexes	[16]
Rat, adult-derived cells	Seminiferous tubules isolated from adult rats after EDS-mediated Leydig-cell depletion	PDGF-AA or PDGF-BB treatment; EdU incorporation and testosterone production during stem Leydig-cell proliferation and differentiation	Both ligands stimulated early stem Leydig-cell proliferation. PDGF-AA promoted subsequent steroidogenic differentiation, whereas sustained PDGF-BB exposure suppressed differentiation and testosterone production	Direct ex vivo reproductive-tissue evidence. Does not establish an adult in vivo requirement; the inhibitory PDGF-BB response was associated predominantly with PDGFRβ	[17]
Mouse, fetal	*Pdgfra*-null fetal XY gonads, fetal gonadal organ culture, and cell-based perturbation models	Genetic and experimental disruption of PDGFRα–ERK signaling; analysis of EGR1, CREB, cord formation, and steroidogenic differentiation	PDGFRα–ERK–EGR1 signaling contributed to morphogenetic events associated with cord formation, whereas PDGFRα–ERK–CREB signaling promoted the fetal Leydig-cell steroidogenic program	Direct genetic and mechanistic developmental evidence. Applicability to adult testicular physiology remains undetermined	[28]
Mouse, adult, 12 weeks	Heterozygous PDGFRα–EGFP reporter testes	Reporter fluorescence, PDGFRα immunostaining, and co-localization with testicular cell-associated markers	PDGFRα-associated signals were detected predominantly in the interstitium, with reported localization in Leydig cells and cells within the peritubular compartment	Adult reproductive-tissue localization evidence. Reporter activity and marker co-localization do not establish receptor activation, definitive lineage identity, or functional coupling	[29]
Human, fetal, and adult	Fetal testes at 16–28 gestational weeks; adult testes with preserved or impaired spermatogenesis; Leydig-cell tumors	Northern blotting, RT-PCR, and immunohistochemical localization of PDGF-A, PDGF-B, PDGFRα, and PDGFRβ	PDGF ligands and receptors exhibited developmental-, cell-, and disease-associated differences in expression and localization	Direct human expression and localization evidence. The descriptive design does not establish receptor activation or causal function	[30]
Mouse and rat Leydig-cell models	TM3 and other Leydig-cell lines; adult mouse testis and primary immature rat Leydig cells used for expression assessment	Rat *Pdgfra* promoter analysis, mutagenesis, DNA-binding assays, and SP1/SP3 overexpression	SP1 and SP3 bound to and activated the *Pdgfra* promoter in TM3 cells	Direct cell-based transcriptional evidence. Predominantly a promoter study in a cell line and does not demonstrate regulation of PDGFRα signaling or reproductive function in vivo	[31]
Mouse, fetal to adult; Leydig-cell model	Mouse testes across developmental stages and MLTC-1 Leydig cells	FOXA3 localization, *Pdgfra* promoter assays, DNA-binding analyses, and FOXA3-deficient MLTC-1 cells	FOXA3 was strongly expressed in adult Leydig cells and bound to and activated the *Pdgfra* promoter in MLTC-1 cells	Tissue-localization and cell-based transcriptional evidence. Does not establish that FOXA3-dependent regulation of *Pdgfra* changes adult testicular function	[32]
Human, adult	PDGFRα-positive interstitial cells isolated from testicular tissue from four donors; in vitro differentiation and transplantation into LuRKO mice	FACS isolation, mesenchymal and Leydig-directed differentiation, steroid measurements, and xenotransplantation	The isolated cells displayed mesenchymal multipotency and acquired some steroidogenic characteristics, but did not produce detectable testosterone or restore circulating testosterone after transplantation	Direct human cell-based and transplantation evidence. Limited donor number, cell expansion before sorting, and failure of long-term engraftment constrain interpretation	[33]
Human, adult	Normal peritubular and intertubular stromal compartments	Histology, transmission electron microscopy, and immunophenotyping for CD34, PDGFRα, CD31, c-KIT, and α-SMA	CD34-positive/PDGFRα-positive telocytes formed networks in the outer peritubular layer and intertubular stroma around Leydig cells and vessels. PDGFRα immunoreactivity was also detected in CD34-negative/α-SMA-positive myoid cells/myofibroblasts in the inner peritubular layer	Cell-resolved anatomical and ultrastructural evidence. PDGFRα was not specific to telocytes, and no receptor-activation or functional perturbation experiments were performed	[34]
Mouse cell line	TM3 Leydig cells with intact or experimentally disrupted primary cilia	PDGF-AA stimulation, PDGFRα localization, AKT/ERK phosphorylation, and depletion of IFT88 or CEP164	PDGF-AA activated AKT and ERK around the ciliary base and promoted TM3-cell growth, migration, and invasion; disruption of ciliary proteins attenuated these responses	Direct cell-line signaling evidence. Does not establish physiological function in primary Leydig cells or intact adult testis	[36]
Rat, prenatal exposure with neonatal assessment	Maternal exposure to DES, BPA, genistein, or coumestrol from gestational day 14 to birth; neonatal testes and purified gonocytes	Testicular *Pdgfra/Pdgfrb* mRNA, in situ hybridization, and PDGFRα/PDGFRβ protein analyses	Estrogenic exposures produced dose-, receptor-, and compartment-dependent changes in receptor transcript abundance and protein distribution	Direct reproductive-tissue exposure evidence. Expression changes do not establish receptor phosphorylation, signaling direction, or functional consequences	[37]
Rat, early postnatal	Imatinib treatment on postnatal days 5–7 with short- and long-term testicular assessment	Pharmacological inhibition of PDGFR, KIT, and ABL-family kinases	Imatinib reduced type-A spermatogonial and mesenchymal myoid-precursor proliferation, increased germ-cell apoptosis, and shortened seminiferous cords; adult epididymal sperm counts recovered	In vivo reproductive pharmacological evidence. Effects cannot be attributed specifically to PDGFRα because imatinib inhibits multiple kinases	[39]
Rat, early postnatal exposure with adult follow-up	Imatinib treatment on postnatal days 5–7, followed by endocrine, histological, and mating analyses at adulthood	Broad tyrosine kinase inhibition; adult gonadotropins, testicular testosterone, histology, gene expression, and reproductive outcomes	Early treatment reduced adult litter size and caused modest persistent gonadotropin elevation despite recovery of testicular morphology and SCF/KIT and PDGF/PDGFR transcript levels	Longitudinal in vivo functional evidence. Nonselective inhibition prevents attribution to PDGFRα, and the mechanism underlying the reduced litter size was not resolved	[40]
Human, adult	Corpus cavernosum from three men with normal erection and five patients with organic erectile dysfunction, including diabetic and non-diabetic ED	Single-cell RNA sequencing, anatomical validation, ligand–receptor inference, and selected cell-based functional assays	The study defined fibroblast, endothelialcell, and smoothmusclecell heterogeneity and identified disease-associated changes in stromal–vascular communication and Wnt/Hippo-related remodeling	Human cell-resolved disease evidence. PDGFRA served primarily as a fibroblast marker; PDGFRα activation and causal function were not tested	[42]
Mouse, adult, and aging	Corpus cavernosum perivascular fibroblasts	Single-cell profiling, genetic targeting, optogenetic fibroblast depolarization, fibroblast-specific Notch manipulation, and chemogenetic modulation of erection frequency	Perivascular fibroblasts supported vasodilation by reducing norepinephrine availability. Recurrent erection suppressed Notch signaling and increased fibroblast abundance, whereas aging reduced fibroblast numbers and penile blood perfusion	Direct in vivo fibroblast-functional evidence. The study establishes fibroblast and Notch-dependent regulation of penile blood flow, but does not demonstrate dependence on PDGFRα signaling	[43]
Rat, adult disease models; complementary human data	Rat DMED and bilateral CNIED models; human corpus-cavernosum single-cell and tissue characterization	Single-cell RNA sequencing, spatial transcriptomics, multiplex immunofluorescence, CytoTRACE, and gene-set analyses	A PDGFRα-positive/Sca-1-positive fibroblast subset was depleted in experimental ED and localized near vascular structures. Computational analyses suggested angiogenic and endothelial/smoothmuscle differentiation potential	Cell-resolved preclinical and human-associated evidence. Differentiation was inferred computationally and supported by co-localization rather than demonstrated by lineage tracing or functional rescue; PDGFRα-specific signaling was not tested	[44]
Mouse, prepubertal	BPA exposure beginning on postnatal day 22 for three weeks	Testicular single-cell RNA sequencing and computational ligand–receptor analysis	BPA exposure was associated with reduced inferred PDGFA–PDGFRA communication involving Leydig cells and pachytene spermatocytes, despite an overall increase in the number and strength of intercellular interactions	Cell-resolved transcriptomic association. Does not measure ligand binding, PDGFRα protein abundance, receptor phosphorylation, signaling flux, or causality	[57]
Mouse, fetal to adult and aging/injury	TCF21 lineage tracing, in vitro differentiation, normal aging, and EDS-associated testicular injury	Single-cell characterization, inducible lineage tracing, cell sorting, transplantation, and differentiation assays	TCF21-lineage mesenchymal cells contributed to multiple somatic lineages during development and replenished adult somatic populations during aging and after injury; the population expressed *Pdgfra* among several mesenchymal markers	Direct TCF21-lineage evidence but indirect PDGFRα evidence. The population expressed *Pdgfra* among several mesenchymal markers, but PDGFRα signaling was not perturbed or shown to mediate regeneration.	[58]
Mouse, adult	Continuous low-dose-rate radiation at 0.39, 1.29, or 3.46 mGy/h for 21 days	Bulk testicular *Pdgfra* and *Acta2* mRNA; histological, ROS/RNS, TUNEL, comet, and collagen analyses	Collagen deposition increased dose-dependently across the exposed groups. Germ-cell apoptosis increased at 1.29 and 3.46 mGy/h, whereas significant ROS elevation, DNA damage, and increased *Pdgfra* and *Acta2* mRNA were detected primarily at 3.46 mGy/h	Direct reproductive-tissue association. Bulk terminal measurements cannot identify the responding cell population or establish increased PDGFRα protein abundance, receptor activation, or causal involvement in fibrosis	[59]
Rat, postnatal stem/progenitor Leydig-cell models	Seminiferous-tubule-associated stem Leydig cells and progenitor Leydig cells	PDGF-BB treatment; PDGFRβ, MEK/ERK, and PI3K inhibition; proliferation and steroidogenic-gene analyses	PDGF-BB promoted proliferation and suppressed steroidogenic differentiation through PDGFRβ-dependent MAPK and PI3K pathways	Direct reproductive cell-based PDGFRβ evidence. Relevant to the broader PDGF–PDGFR system, but does not provide evidence for PDGFRα activation	[60]

Note: Evidence categories describe what each study directly demonstrated and do not represent a formal risk-of-bias or quality assessment. Expression, cell-based, broad pharmacological, and transcriptomic inference studies do not independently establish a PDGFRα-specific in vivo function. BPA, bisphenol A; CNIED, cavernous nerve injury-induced erectile dysfunction; DES, diethylstilbestrol; DMED, diabetes mellitus-induced erectile dysfunction; ED, erectile dysfunction; EDS, ethane dimethanesulfonate; PDGF, platelet-derived growth factor; PDGFR, platelet-derived growth factor receptor; Sca-1, stem cell antigen-1.

**Table 2:** Evidence-based and proposed roles of PDGFRα-associated signaling in male reproductive health and injury.

Context	Predominant Compartment	PDGFR-Associated Function or Alteration	Reproductive Outcome or Implication	Evidence Basis
Fetal testis development	Interstitial mesenchymal progenitors, fetal Leydig lineage, and other testicular somatic cells	Testis-cord formation and fetal Leydig-cell differentiation	Proper fetal testis morphogenesis and establishment of the steroidogenic lineage	Direct genetic and mechanistic mouse evidence [15,28], supported by *Pdgfa*-deficient mouse findings [16] and human developmental expression/localization evidence [30]
Adult testicular stromal populations	Leydig-lineage and heterogeneous PDGFRα-positive interstitial populations	Candidate roles in progenitor regulation, stromal organization, and injury-associated regeneration	Potential contribution to steroidogenic and spermatogenic support	Ex vivo and cell-characterization evidence [17,29,32,33], cell-based transcriptional and signaling evidence [31,36], and indirect lineage evidence [58]; an adult PDGFRα-specific in vivo requirement for homeostasis, steroidogenesis, fertility, or regeneration has not been established
Cavernosal homeostasis and erectile physiology	Cavernosal and perivascular fibroblast populations	Paracrine regulation of vasoconstriction, penile blood flow, and local tissue homeostasis	Maintenance of erectile function	Direct fibroblast-functional evidence in mouse corpus cavernosum [43] and human cell-resolved anatomical evidence [42]; PDGFRα-specific signaling was not tested
Erectile dysfunction-associated cavernosal remodeling	Fibroblast, endothelial, and smooth-muscle compartments, including PDGFRα-positive/Sca-1-positive fibroblasts	Altered stromal–vascular communication and depletion of a putative angiogenic or vascular-repair-associated fibroblast population	Endothelial and smooth-muscle loss, impaired cavernosal perfusion, and erectile dysfunction	Human cell-resolved disease association [42] and rat diabetes mellitus-associated and cavernous nerve injury-associated erectile dysfunction evidence [44]; differentiation potential and PDGFRα-specific causality remain unproven
Prenatal estrogenic perturbation	Neonatal rat testicular interstitium and seminiferous cords	Cell- and receptor-dependent changes in *Pdgfra* and *Pdgfrb* mRNA abundance and PDGFRα/PDGFRβ protein distribution	Evidence of developmental perturbation of the testicular PDGFR system; functional consequences remain undetermined	Direct reproductive-tissue expression and localization evidence [37]; receptor activation and causal reproductive outcomes were not assessed
Early postnatal broad-spectrum tyrosine kinase inhibition	Spermatogonial/germ-cell, mesenchymal myoid/peritubular precursor, and Leydig-lineage/endocrine compartments	Reduced spermatogonial and mesenchymal myoid-precursor proliferation, increased germ-cell apoptosis, and impaired seminiferous-cord development after imatinib exposure	Transient developmental disruption with persistent gonadotropin elevation and reduced adult litter size despite recovery of sperm count, testicular morphology, and ligand–receptor mRNA abundance	Direct reproductive-tissue pharmacological evidence [39,40], supported by a scoping review [41], because imatinib also inhibits KIT, ABL-family kinases, and PDGFRβ, these effects cannot be attributed specifically to PDGFRα
Prepubertal BPA exposure	Leydig cells and pachytene spermatocytes within the pubertal mouse testis	Reduced inferred PDGFA–PDGFRA communication involving specific somatic and germ-cell populations	Association with BPA-induced testicular injury and possible disruption of somatic–germ-cell communication	Cell-resolved reproductive-tissue evidence based on single-cell transcriptomic ligand–receptor inference [57]; PDGFRα protein abundance, receptor phosphorylation, and causal function were not measured
Chronic low-dose-rate radiation	Cellular origin unresolved in the bulk adult testis	Increased bulk testicular *Pdgfra* mRNA associated with oxidative injury and fibrosis	Possible repair response, stromal expansion, or maladaptive remodeling	Direct reproductive-tissue association [59]; receptor activation and cellular source unresolved

Note: This table integrates directly observed findings with proposed biological interpretations. Terms such as “candidate,” “potential,” and “possible” indicate hypotheses that have not been demonstrated causally. Evidence based on expression or localization, cell-based assays, broad pharmacological inhibition, or transcriptomic ligand–receptor inference does not independently establish a PDGFRα-specific in vivo function. BPA, bisphenol A; PDGF, platelet-derived growth factor; PDGFR, platelet-derived growth factor receptor; Sca-1, stem cell antigen-1.

## Data Availability

No new data were created or analyzed in this study. Data sharing does not applicable to this article.

## Abbreviations

The following abbreviations are used in this manuscript:

ABL	Abelson tyrosine kinase
AKT	Protein kinase B
ANO1	Anoctamin 1
BPA	Bisphenol A
CREB	cAMP response element-binding protein
DEHP	Di(2-ethylhexyl) phthalate
EGF	Epidermal growth factor
EGFR	Epidermal growth factor receptor
EGR1	Early growth response protein 1
ERK	Extracellular signal-regulated kinase
FGFR	Fibroblast growth factor receptor
FOXA3	Forkhead box A3
MAPK	Mitogen-activated protein kinase
NADPH	Reduced nicotinamide adenine dinucleotide phosphate
NOX	NADPH oxidase
PDGF	Platelet-derived growth factor
PDGFR	Platelet-derived growth factor receptor
PI3K	Phosphoinositide 3-kinase
PLCγ	Phospholipase C gamma
PPARγ	Peroxisome proliferator-activated receptor gamma
PRISMA	Preferred Reporting Items for Systematic Reviews and Meta-Analyses
PTP	Protein tyrosine phosphatase
Rac1	Ras-related C3 botulinum toxin substrate 1
ROS	Reactive oxygen species
SASP	Senescence-associated secretory phenotype
Sca1	Stem cell antigen-1
SMAD	Suppressor of mothers against decapentaplegic
Sp1	Specificity protein 1
Sp3	Specificity protein 3
TGF-β	Transforming growth factor beta
VEGF	Vascular endothelial growth factor
VEGFR	Vascular endothelial growth factor receptor
